# Resolvin D1‐loaded nanoliposomes promote M2 macrophage polarization and are effective in the treatment of osteoarthritis

**DOI:** 10.1002/btm2.10281

**Published:** 2022-03-07

**Authors:** Ameya A. Dravid, Kaamini M. Dhanabalan, Smriti Agarwal, Rachit Agarwal

**Affiliations:** ^1^ BioSystems Science and Engineering Indian Institute of Science Bangalore Karnataka India

**Keywords:** inflammatory diseases, nanocarriers, regenerative medicine, resolution of inflammation, specialized proresolution mediators

## Abstract

Current treatments for osteoarthritis (OA) offer symptomatic relief but do not prevent or halt the disease progression. Chronic low‐grade inflammation is considered a significant driver of OA. Specialized proresolution mediators are powerful agents of resolution but have a short in vivo half‐life. In this study, we have engineered a Resolvin D1 (RvD1)‐loaded nanoliposomal formulation (Lipo‐RvD1) that targets and resolves the OA‐associated inflammation. This formulation creates a depot of the RvD1 molecules that allows the controlled release of the molecule for up to 11 days in vitro. In surgically induced mice model of OA, only controlled‐release formulation of Lipo‐RvD1 was able to treat the progressing cartilage damage when administered a month after the surgery, while the free drug was unable to prevent cartilage damage. We found that Lipo‐RvD1 functions by damping the proinflammatory activity of synovial macrophages and recruiting a higher number of M2 macrophages at the site of inflammation. Our Lipo‐RvD1 formulation was able to target and suppress the formation of the osteophytes and showed analgesic effect, thus emphasizing its ability to treat clinical symptoms of OA. Such controlled‐release formulation of RvD1 could represent a patient‐compliant treatment for OA.

## 1 INTRODUCTION

According to recent estimates, 303.1 million patients were suffering from osteoarthritis (OA) in 2020 worldwide.^1^ Despite the widespread prevalence of the disease, there are no approved disease‐modifying OA drugs (DMOADs) for human use. OA is characterized by progressive loss of cartilage, pain, damage to the subchondral bone, and eventual loss of function of the affected joint in humans.^2^ Current treatment includes administering glucosamine, glucocorticoids, and other NSAIDS but are mainly targeted toward symptomatic relief.^3^, ^4^, ^5^ These treatments often fail to arrest the progressing cartilage deterioration and have several other drawbacks like gastric bleeding and increased propensity to osteoporosis in women.^6^, ^7^ Most OA patients eventually require highly invasive and expensive joint‐replacement surgery. Other strategies, like viscosupplementation and oral glucosamine administration, have achieved inconclusive results in humans.^8^, ^9^ Due to the lack of a reliable DMOAD, tremendous loss of quality of life and revenue goes unchecked annually.

Factors like old age, diet, obesity, and trauma contribute to inflammation in humans. Chondrocyte viability is reduced, resulting in altered matrix synthesis, high levels of proinflammatory cytokines (interleukin‐1β [IL‐1β] and tumor necrosis factor‐α [TNF‐α]), and production of catabolic enzymes.^10^ This chronic, low‐grade inflammation is a significant driver of OA and is reflected in the surge in the levels of inflammatory cytokines in the synovial fluid and systemic circulation.^11^, ^12^ Thus, blockade of inflammation by inhibiting the action of inflammatory cytokines like IL‐1β and TNF‐α is considered a viable treatment strategy for OA.^13^, ^14^, ^15^ However, such approaches have proved subtherapeutic in human clinical trials.^13^, ^14^ This failure is attributed to the efficient lymphatic drainage that rapidly clears (1–5 h) off the therapeutic molecules from the joint. Some therapeutics like tanezumab (antibody against nerve growth factor) reduce pain in the short term but fail to cease damage to the cartilage.^16^

M1 macrophages are known to be a major source of proinflammatory, cartilage‐damaging cytokines in OA.^17^, ^18^ Such dysfunction arises due to impaired proresolution programs at the disease site.^19^, ^20^ These programs are endogenous cellular pathways and activities that inhibit the infiltration of more immune cells and coordinate the postinflammatory clearance of inflammatory cells and other debris.^21^, ^22^, ^23^ These activities are coordinated by a class of molecules called specialized proresolution mediators (SPMs).^24^ Resolvins are one such class of molecules that regulate the proinflammatory activities of aggressor cells and eventually block the progressing damage. They are powerful agents acting at cellular levels and can potentially break the cycle of chronic inflammation.^25^, ^26^, ^27^ One species of highly bioactive SPMs, Resolvin D1 (RvD1), is known to polarize macrophages to a proresolution M2 phenotype instead of the M1 phenotype.^28^, ^29^ RvD1 is versatile in its activity, and mediates clearance of debris,^30^ reduces the influx of phagocytes,^31^ and promotes anabolism in chondrocytes.^32^ Although RvD1 is shown to have increased expression in OA joints, its levels are not sufficient to drive tissue healing.^33^ Exogenously administered RvD1 reduces the severity of OA in rodents,^29^ but the short half‐life and limited in vivo retention of such molecules limits their therapeutic potential.

Since small‐molecule drugs diffuse rapidly out of the joint, their intra‐articular (IA) delivery has not been successful in the treatment of joint‐related diseases.^34^, ^35^ The use of particle‐based drug carriers can increase the effective half‐life of drugs.^36^, ^37^ Lipid‐based drug delivery systems like liposomes are ideal drug carriers because of their biodegradability, low toxicity, stability, flexible synthesis methods, and ability to incorporate versatile cargo (imaging agents, corticosteroids, MMP inhibitors).^38^, ^39^, ^40^ Such systems have also shown excellent translational potential and have resulted in several clinically approved treatments such as Doxil®, Ambisome®, and DaunoXome. Liposomes have been used for the IA delivery of corticosteroids.^41^, ^42^ One such liposomal formulation, Lipotalon®, has been approved in Germany for treating OA patients. The active ingredient of Lipotalon®, palmitoylated dexamethasone, suppresses inflammation by preventing the infiltration of neutrophils and reducing the proliferation of leukocytes.^43^ In this study, we have developed RvD1‐encapsulated liposomes for IA delivery in OA (provisional Indian patent application number: 202141040859). The liposomes were stable and nontoxic and were retained for substantially longer durations in the joints compared to the free drug. Lipo‐RvD1 was effective in preventing OA when administered as prophylactic as well as therapeutic regimen. The mechanistic analysis showed that the ratio of M1/M2 cells decreases with the administration of Lipo‐RvD1, leading to reduced inflammatory and catabolic markers such as MMP13 and ADAMTS5.

## 2 EXPERIMENTAL SECTION

### 2.1 Materials

The lipids dipalmitoylphosphatidylcholine (DPPC), 1, 2‐distearoyl‐sn‐glycero‐3‐phosphoethanolamine–poly (ethylene glycol) (DSPE–PEG), cholesterol, dioleoyl‐3‐trimethylammonium propane (DOTAP) were purchased from Avanti polar lipids. RvD1 was purchased from Cayman chemicals. Syringes were purchased from BD Biosciences. Antibodies used: anti‐iNOS (NB300‐605) and anti‐CD206 (NBP1‐90020) antibodies were purchased from Novus Biologicals (Centennial). Anti‐ADAMTS5 (ab41037) and anti‐MMP13 (ab39012) antibodies were purchased from Abcam. Solvents: acetonitrile, methanol, and HPLC grade water were purchased from Fisher Chemicals. All purchased compounds were used without further purification.

### 2.2 Quantification of RvD1

RvD1 was quantified using Agilent 1200 or Shimadzu Prominence‐i HPLC. Briefly, samples containing RvD1 were injected in the RP‐C18 column and eluted using a binary gradient of methanol and water (total flow rate of 0.40 ml/min). The retention time of RvD1 was 46 mins. The area under the curve for the RvD1 peak was used to plot standard curves and quantify RvD1 in experimental samples.

### 2.3 Synthesis and characterization of liposomes

Liposomes were synthesized by the thin‐film lipid hydration method. Briefly, the lipids DPPC, DSPE–PEG, and cholesterol were dissolved in chloroform and mixed in their respective molar ratios in a round bottom flask. The chloroform was evaporated using a rotatory evaporator (DLAB RE100 Pro), thus forming thin lipids films. The films generated were hydrated using respective solutions (AF750 in phospahte‐buffered saline [PBS] for in vivo retention experiments, calcium acetate for all RvD1 loading experiments) at 45°C. The vesicles were then collected and passed through 1 μm, 400 nm, and 100 nm membranes to generate liposomes of a defined size. To test the stability of the liposomes, we incubated the liposomes in PBS for up to 10 days at 37°C. The sizes of liposomes were measured using Malvern Zetasizer μV.

### 2.4 Cryogenic transmission electron microscope of liposomes

Liposomes were imaged using cryogenic transmission electron microscope (Cryo‐TEM). Briefly, Holey Carbon Flat R2/2 grids were blotted with a liposomal solution (2 mg/ml) and plunged into liquid ethane using FEI vitrobot to generate vitrified samples. These samples were stored in liquid nitrogen till further use. Furthermore, images of these samples were captured using Thermo Scientific Arctica equipped with a Gatan K2 direct electron detector camera by Latitude S software with a spot size of 7. The total exposure was 40 e^−^/Å^2^, and the pixel size was 1.2 Å.

### 2.5 Loading of RvD1 into liposomes

RvD1 was loaded into liposomal using a remote‐loading strategy.^44^ Briefly, thin films of lipids (described earlier) were hydrated with 120 mM calcium acetate (pH 6) to generate multilamellar vesicles. These vesicles were extruded through filters of different pore sizes (1 μm, 400 nm, and 100 nm) to generate liposomes of desired sizes. Finally, the liposomes were pelleted and resuspended in RvD1‐containing sodium sulfate solution (pH 4) and loaded at 50°C for 1.5 h. After loading, the formulation was washed twice in PBS and used immediately.

### 2.6 Release profiles

For characterizing the retention of RvD1, liposomes were incubated in PBS for 7 days at 37°C. At each time point, the liposomes were washed and lyophilized. Lyophilized powder was dissolved in 50% isopropanol and the samples were loaded and quantified using HPLC as described earlier.

### 2.7 Mice for in vivo studies

The in vivo studies were approved by the Institutional Animal Ethics Committee (CAF/Ethics/612/2018). Male C57BL/6 mice (aged 6–8 weeks; weight 20–22 g) were used for this study, maintained in individually ventilated cages at the central animal facility, Indian Institute of Science, Bangalore. The animals were allowed access to feed and water ad libitum. A cocktail of ketamine (60 mg/kg) and xylazine (9 mg/kg) was used to anesthetize animals during procedures.

### 2.8 In vivo retention of liposomes on cartilage

Liposomes for injections were synthesized, as discussed earlier. The thin film of lipids was hydrated with Alexa Fluor 750 (AF750, 400 μg/ml) solution. Liposomes (~1 mg per joint, in a total volume of 10 μl) were injected intra‐articularly in the knee joint of mice. The fluorescence intensity was captured using Bruker XTreme II at excitation/emission 750/830 nm and analyzed using Bruker molecular imaging software.

### 2.9 Mice model of OA

We used a surgical model, destabilization of the medial meniscus (DMM), to induce OA in male mice.^45^, ^46^ The experiments utilized male mice because surgery‐induced OA show more dramatic effects on the cartilage of male mice than that of female mice.^47^ One knee joint was subjected to this surgery in each mouse. Briefly, the mice were anesthetized using a cocktail of ketamine (60 mg/kg) and xylazine (9 mg/kg). After confirming the loss of pedal reflexes, a parapatellar skin incision was made to access the synovium. The synovium was then dissected to expose the underlying joint. The medial meniscotibial ligament (MMTL) was located and surgically transected. After confirming the successful transection of the MMTL by another observer, the synovium and skin were sutured in layers, and a metronidazole wet pack was applied over the sutured area. The mice were then allowed to recover from the anesthesia on a lukewarm surface. Postoperative analgesic care included four subcutaneous doses of buprenorphine (0.1 mg/kg), once every 12 h. Later, the mice were administered their respective IA treatments over the next 3 months, as indicated in the result section. The mice were allowed to move freely for the entire duration of the study. At the end of the study, the mice were euthanized, and their knee joints were harvested. The joints were fixed in 4% formaldehyde for 6 h and decalcified in 5% formic acid for 5 days. Following decalcification, the joints were dehydrated in multiple gradients of ethanol and xylene and finally embedded in paraffin wax.

### 2.10 Histology and staining

The tissues embedded in paraffin blocks were sectioned into 5 μm thick sections using Leica HistoCore MULTICUT and collected on poly‐l‐lysine‐coated glass slides. The sections were hydrated using a series of ethanol gradients and stained using the Safranin O.^48^ The severity of the disease was quantified on a scale of 0–24 using a scoring protocol prescribed by OARSI.^49^ This protocol considers the joint's damage, including loss of proteoglycan, chondrocyte apoptosis, and the presence of osteophytes.^49^ Scoring was done by trained veterinarians blinded to the study.

### 2.11 Immunohistochemistry

Immunohistochemistry (IHC) was performed to quantify iNOS^+^ cells CD206^+^ cells. MMP13^+^ and ADAMTS5^+^ regions in the synovial membrane and cartilage. Heat‐induced epitope retrieval was performed using Tris‐EDTA overnight treatment at 65°C, followed by retrieval with 1 N HCl and Trypsin‐CaCl_2_. The resulting sections were then incubated with primary antibody for 16 h. These sections were then washed to remove excess unbound antibody and incubated with horseradish peroxidase‐conjugated secondary antibody for 2 h. After washing the unbound secondary antibody, sections were incubated with 3,3′‐diaminobenzidine (DAB) substrate for 1 h. Excess unreacted DAB was washed, and images were captured using an Olympus BX53F brightfield microscope. The images were thresholded against the background and counted using the “analyze particles” module in ImageJ.

### 2.12 Testing for mechanical allodynia

OA‐associated allodynia was tested using von Frey filaments (Aesthesio tactile sensory filament; Ugo Basile). Briefly, the animals were introduced in a customized cage with perforated bottom (Figure S1) and were allowed to be acclimated to it for a few minutes. A von Frey filament representing the smallest force was pushed against the plantar regions of the paw of respective animals through the perforations in the cage. This exercise was performed five times per filament. If the animal withdrew its paw three out of the five times, the force represented by that filament was noted as the paw withdrawal threshold. If no such activity was seen, the exercise was continued with the next filament. Only animals with no visible injury were used for data collection.

### 2.13 MicroCT experiments

MicroCT was performed on joints using Zeiss Xradia versa 500. Briefly, the joint was mounted in the instrument and 750 projections were acquired at source voltage 10 V, power 140 W, and voxel size 10 μm. The data were analyzed using the 3DSlicer software. A region of interest (ROI) (representing the bone to be analyzed) was carefully cropped from the subchondral bone of the medial tibia into an independent object. The dimensions of the ROI were kept constant for all the subsequently analyzed joints. In this object, after setting a threshold to differentiate the bone from its background, the ComputeBMFeatures module was used to calculate the bone morphometric parameters.

### 2.14 Statistics

Data presented in this manuscript were represented as mean ± standard deviation with at least three replicates in each group unless stated otherwise. Data were analyzed using one‐way analysis of variance for normal distributions and using other nonparametric tests (e.g., Dunn's or Kruskal–Wallis tests) for ordinal datasets. Outliers were analyzed using the Grubbs test (*α* = 0.05). The 95% confidence interval was considered significant.

## 3 RESULTS

### 3.1 Submicron‐sized liposomes were synthesized from inert, biocompatible lipids

**FIGURE 1:**
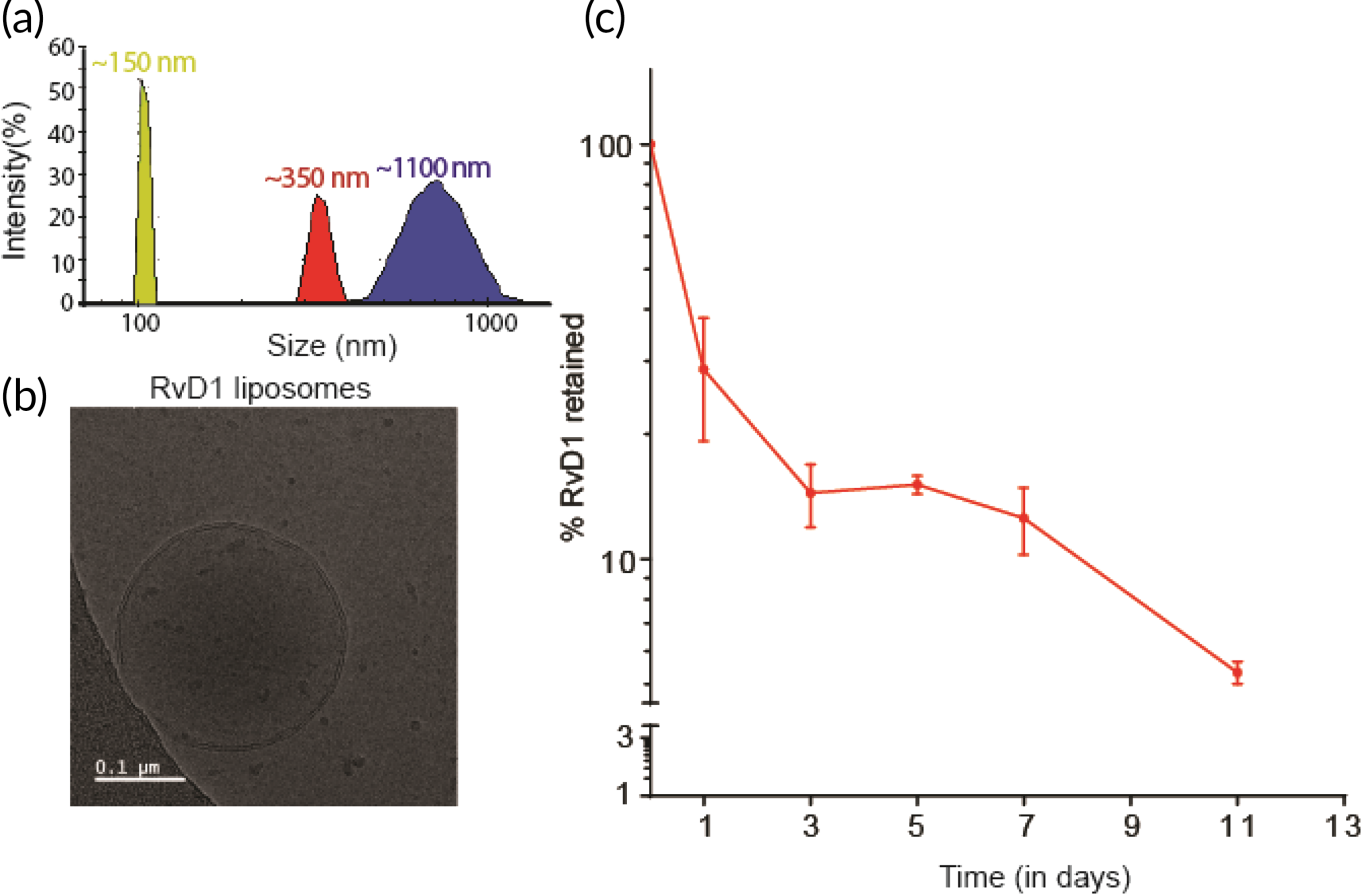
Characterization and release profile of Lipo‐RvD1. (a) The size distribution of liposomes used in this study as measured using dynamic light scattering. (b) Cryo‐TEM micrographs of Lipo‐RvD1. (c) Quantification of in vitro release of RvD1 from Lipo‐RvD1 when incubated at 37°C at pH 7.4; *n* = 3. Cryo‐TEM, cryogenic transmission electron microscope

Liposomes loaded with the active molecules act as a depot that can release the cargo at therapeutic concentrations in a controlled manner.^15^, ^36^, ^50^ We synthesized liposomes by hydrating thin films of lipids and extruding the resulting multilamellar vesicles through porous membranes with pore sizes 100, 400, and 1000 nm. The sizes and morphology of particles were then analyzed using dynamic light scattering (Figure 1a) and cryo‐TEM (Figure 1b). The sizes of liposomes obtained finally were around 150, 350, and 900 nm. We had chosen these sizes because particles in these size ranges were previously shown to have long IA retention times.^51^ Two different sizes of liposomes were tested for stability (150 and 700 nm) and were found to be stable in PBS for >10 days (Figure S2).

### 3.2 RvD1 can be loaded efficiently using the active‐loading approach

Loading of the anti‐inflammatory ω‐3 fatty acids into biomaterials for controlled release is a viable strategy to treat inflammatory diseases. RvD1 had been shown to successfully reduce the influx of neutrophils after delivery via poly‐l‐(lactic‐co‐glycolic acid) scaffolds.^52^ At a tissue level, resolvin E1 delivery via polymeric nanoparticles successfully treated intestinal wounds in mice.^53^ While successful, these strategies are not amenable for IA delivery due to low encapsulation efficiencies or large size. We initially loaded RvD1 passively by hydrating dry films of lipids with 1 ml of 1 μg/ml RvD1 solution. We found that the encapsulation efficiency when RvD1 was loaded passively (determined using HPLC; Figure S3) was <1%. To overcome this challenge of low loading, we then loaded RvD1 into liposomes actively by employing a differential pH gradient across the lipid bilayer to drive the RvD1 molecule into the intraliposomal space. This strategy was successful, and we were able to achieve the encapsulation efficiency of 71 ± 28% (with loading levels of 35.7 ± 16.15 ng/mg of lipid). In addition, RvD1 loading in liposomes was tunable, and we were able to load RvD1 at various different levels with high encapsulation efficiencies (up to 1065 ± 92 ng RvD1/mg liposomes) (Figure S4). While there have been previous attempts to load RvD1 in liposomes,^54^, ^55^, ^56^ to our knowledge, this is the first report of active loading of RvD1 into liposomes and results in much higher encapsulation efficiencies than any previous report. Since RvD1 is extremely potent and is shown to work at picomolar and nanomolar ranges,^57^ we used Lipo‐RvD1 with lower loading (35.7 ± 16.15 ng/mg of lipid) for our subsequent experiments.

The intraliposomal retention of small‐molecule drugs over time directly correlates with their composition, especially cholesterol concentration.^58^, ^59^, ^60^ Several groups have shown that the cholesterol levels in the liposomes trigger the rapid release of the encapsulated molecules.^61^, ^62^ This is because the cholesterol molecules intercalate between the long tails of other lipids and increase the fluidity and permeability of the bilayer. We found that while the percentage of cholesterol in the lipid formulations does not affect RvD1 loading (Figure S5), 10% of cholesterol formulations show slower release than other formulations containing higher amounts of cholesterol (Figure S6). As lower than 10% cholesterol in liposomes are known to cause instability,^61^, ^63^ for our future experiments, we used 10% cholesterol in our lipid formulations.

To test the temporal release in vitro, Lipo‐RvD1 was incubated in PBS at 37°C for various time intervals. Drug remaining in the liposomes was quantified using HPLC. We found that the RvD1 molecules were retained intraliposomally for >11 days (Figure 1c).

### 3.3 Liposomes increase IA retention of loaded molecules

**FIGURE 2:**
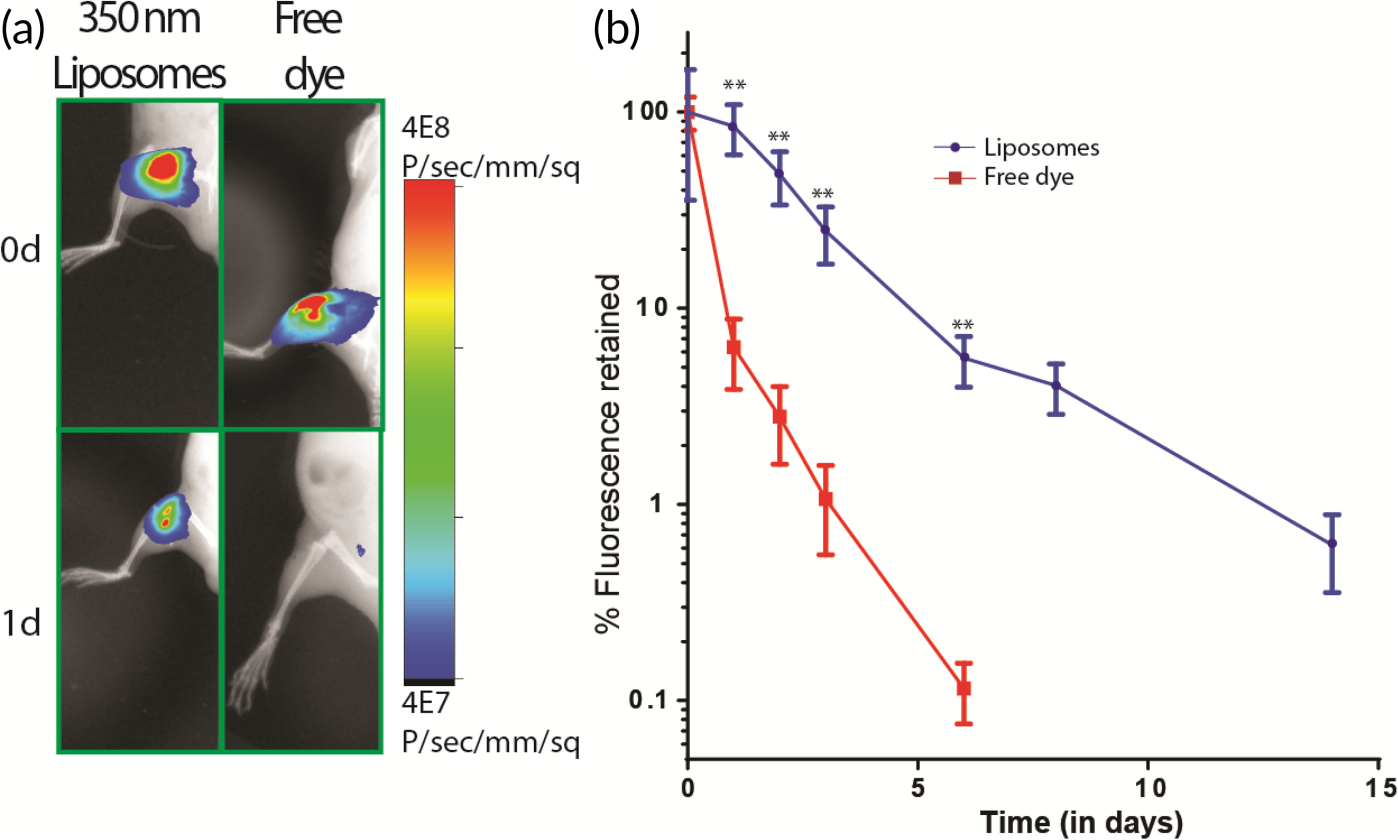
Liposomes are retained longer than free dye in the respective joint. (a) Fluorescence images of live mice depicting the difference between IA retention of fluorescent liposomes and free dye at Day 0 and Day 1. (b) Quantification of retention of IA‐injected fluorescent liposomes and free dye from the respective joint; for liposome‐injected joints, *n* = 4 and for free dye‐injected joints, *n* = 5. **p < 0.01 between free drug and liposomes using unpaired *t*‐test at respective time points. Values are expressed as mean ± *SEM*. IA, intra‐articular

To test whether our liposomal formulations had high retention in vivo, we synthesized AF750‐loaded fluorescent liposomes. This dye allowed for sensitive quantification of fluorescence through live tissues since its emission spectrum has little overlap with tissue autofluorescence. Our data showed that IA‐injected liposomes had significantly higher retention than free dye from Day 1 onwards. While more than 90% of the free dye was cleared within 1 day, liposome‐encapsulated dye signal was present even after 14 days (Figure 2a,b). This difference in the temporal retention is expected because the joint can efficiently clear small molecules via lymphatic clearance, but not large particles.^34^ It is important to note that the signal measured, represent the dye remaining in the joint and it is possible that the liposomes were retained for longer duration.

The size of the carriers are known to have an important effect on their IA retention.^51^, ^64^ We tested IA retention of three different sizes in the range—150, 350, and 900 nm. Our results show that smaller liposomes (≤350 nm) had longer retention than larger liposomes (~900 nm) (Figure S7). This could be due to the saturation of phagocytic clearance by synovial macrophages as higher number of smaller particles are present in the same weight of lipids compared to larger‐sized liposomes.^65^ Further experiments are required to understand this trend. For the rest of our studies, we used 350 nm liposomes due to their higher retention in the joint.

Several groups have shown the importance of coulombic attraction between the anionic cartilage and cationic liposomes in IA retention.^66^, ^67^ We synthesized cationic liposomes (*ζ* potential = +8.1 ± 0.27 mV) by adding DOTAP lipid to the lipid mixture used to synthesize liposomes (Figure S8a). The positive charge was capped at 7% DOTAP because highly cationic liposomes are known to be cytotoxic.^68^ Seven percent DOTAP did not show any visible swelling or signs of inflammation in the weeks following IA injections. These liposomes were injected IA and tested for retention. As shown in Figure S8b, both formulations, unmodified (*ζ* potential = −30 ± 0.05 mV) and cationic, showed similar retention. This trend could be because of the presence of PEG on the surface, which can shield the surface charge and inhibit the attractive coulombic interactions between the liposomes and cartilage matrix.^69^ Since the clearance rates of both formulations were similar, and cationic liposomes are also known to cause toxicity and activate the complement system,^70^ we proceeded with plain liposomes for our future experiments.

### 3.4 Lipo‐RvD1 reduce the severity of OA in mice

**FIGURE 3:**
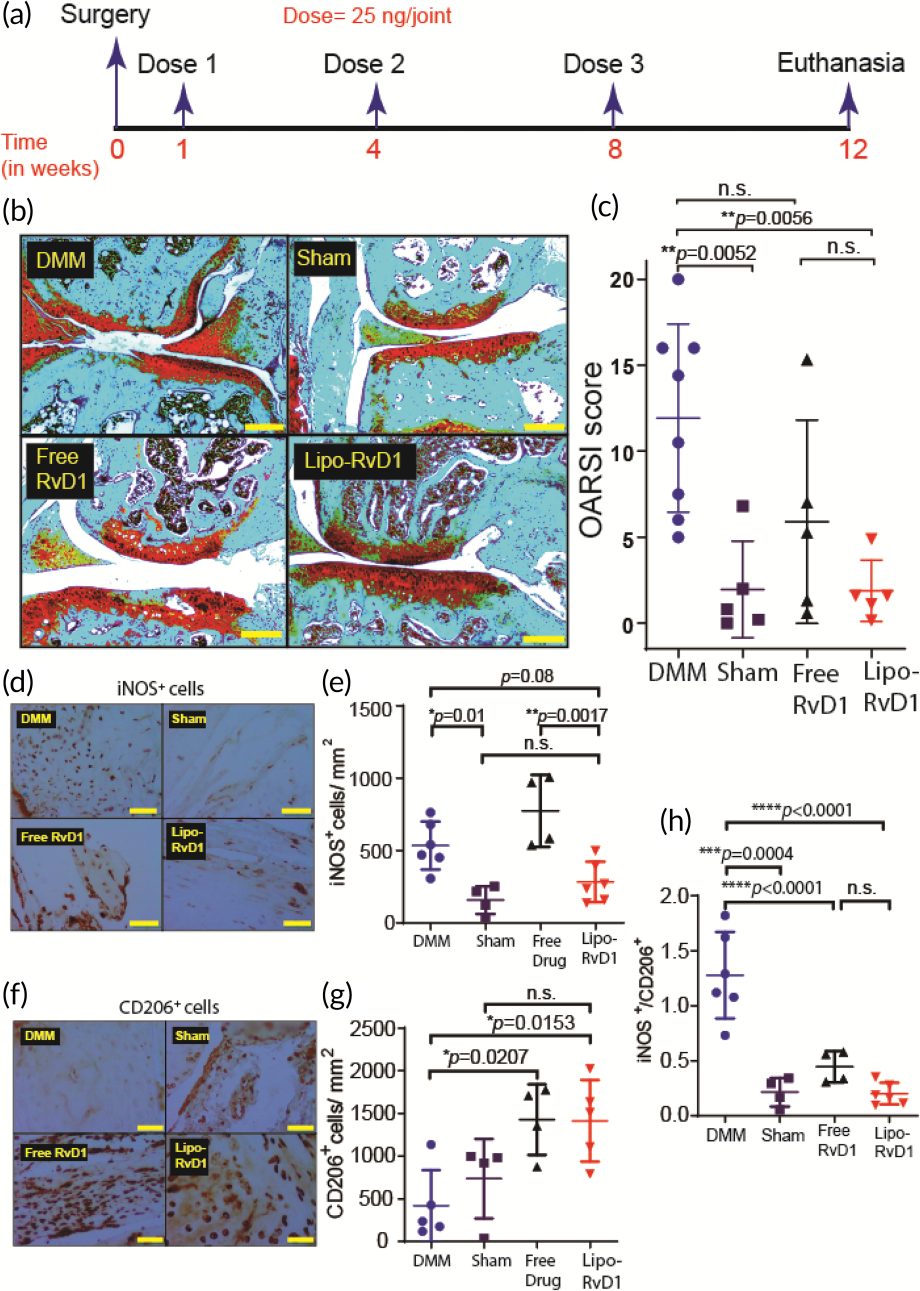
Prophylactic administration of Lipo‐RvD1 alleviates cartilage damage. (a) Timeline of the experiment. (b) Characteristic Safranin O‐stained histology sections of different groups of mice (scale bar ‐ 200 μm). (c) OARSI scores of Safranin O‐stained sections of mice knee joints administered with respective treatment; *n* = 5 sham control joints and joints injected with free RvD1, *n* = 8 DMM joints, and *n* = 6 joints injected with Lipo‐RvD1. (d) IHC images depicting levels of iNOS^+^ M1 macrophages synovial membrane (scale bar = 50 μm). (e) Quantification of iNOS^+^ M1 macrophages in the synovial membrane; *n* = 4 sham control joints and joints treated with free RvD1, *n* = 6 DMM joints and joints treated with Lipo‐RvD1. (f) IHC images depicting levels of CD206^+^ M2 macrophages in synovial membrane (scale bar = 50 μm). (g) Quantification of CD206^+^ M2 macrophages in the synovial membrane; *n* = 4 sham control joints and joints treated with free RvD1, *n* = 5 DMM joints and joints treated with Lipo‐RvD1. (h) The ratio of M1/M2 cells in the synovial membrane; *n* = 4–6 animals per group. For c, one point in the Lipo‐RvD1 group was removed after outlier analysis (Grubbs test). For c, e, g, and h,*p < 0.05, **p < 0.01, ***p < 0.001, and ****p < 0.0001 between the respective groups indicated in the figures using ANOVA followed by Tukey's posthoc test. Values are expressed as mean ± *SD*. ANOVA, analysis of variance; DMM, destabilization of the medial meniscus; IHC, immunohistochemistry

The surgical model of DMM is a reliable model for posttraumatic OA, which is prevalent in 12%–15% of all OA patients.^71^ The medial meniscus is soft fibrocartilage located between the articulating surfaces and absorbs mechanical shock. Surgically cutting this tissue results in the contact between the two articulating surfaces and generates OA‐like changes over 1–3 months.^45^, ^72^ We performed DMM surgery in mice and used a prophylactic dosing regimen by injecting freshly synthesized Lipo‐RvD1 intra‐articularly at weeks 1, 4, and 8 after surgery (Figure 3a). Weight monitoring showed no adverse effects as animals in all the groups continued to gain weight at a steady rate (Figure S9). Our results show that Lipo‐RvD1 could impede OA progression by maintaining the overall joint integrity. Specifically, we observed that the Lipo‐RvD1‐treated mice had a well‐maintained matrix in all the cartilage layers, and showed a sixfold reduction in OARSI scores compared to DMM joints (*p =* 0.0056), which showed severe denudation (Figure 3b,c). Administration of free RvD1 generated a mild protective effect (twofold reduction in OARSI score) on the cartilage but was not significant compared to DMM group. Lipo‐RvD1 showed complete protection of the cartilage with OARSI scores similar to animals that had undergone sham surgery (Figure 3c). Lipo‐RvD1 also showed a threefold reduction in OARSI compared to free RvD1 but was not statistically significant. In addition, the stained sections showed a higher percentage of healthy and nonhypertrophic chondrocytes in Lipo‐RvD1‐treated animals compared to DMM and free RvD1‐treated mice (Figure 3b,c). Several studies have reported the role of M1/M2 macrophage imbalance in inflammatory diseases.^73^, ^74^ The ratio of M1/M2 cells is skewed in OA, and proinflammatory cytokines from M1 drive cartilage damage.^75^ Previously it was shown that administration of RvD1 before induction of damage promotes the presence of M2 macrophages in the synovial membrane.^29^ As seen from our results, DMM mice had higher levels of M1 cells than sham mice (*p* = 0.0052), which is indicative of a proinflammatory phenotype in this disease (Figure 3d,e). Administration of Lipo‐RvD1 reduced the levels of proinflammatory M1 cells as compared to free RvD1 joints (*p =* 0.0017). Furthermore, Lipo‐RvD1 treatment triggered the preferential polarization toward M2 cells as compared to DMM mice (*p* = 0.0153) (Figure 3f,g). Further analysis showed that Lipo‐RvD1 successfully decreased the ratio of M1/M2 cells when compared to DMM joints (*p* < 0.0001) (Figure 3h). Overall, we observed that the formulation was reducing the net inflammatory activity of the synovium by reducing M1 cells and promoting clearance of debris and other inflammatory factors by increasing M2 cells in the joint. Catabolic enzymes like ADAMTS5 and MMP13 are released by chondrocytes in OA and are considered as markers of chondrocyte hypertrophy.^76^ Administration of Lipo‐RvD1 suppresses the expression of ADAMTS5 and MMP13, thus indicating the suppression of hypertrophic chondrocytes phenotype in OA in mice (Figure S10).

### 3.5 Lipo‐RvD1 is a good therapeutic candidate for OA

**FIGURE 4:**
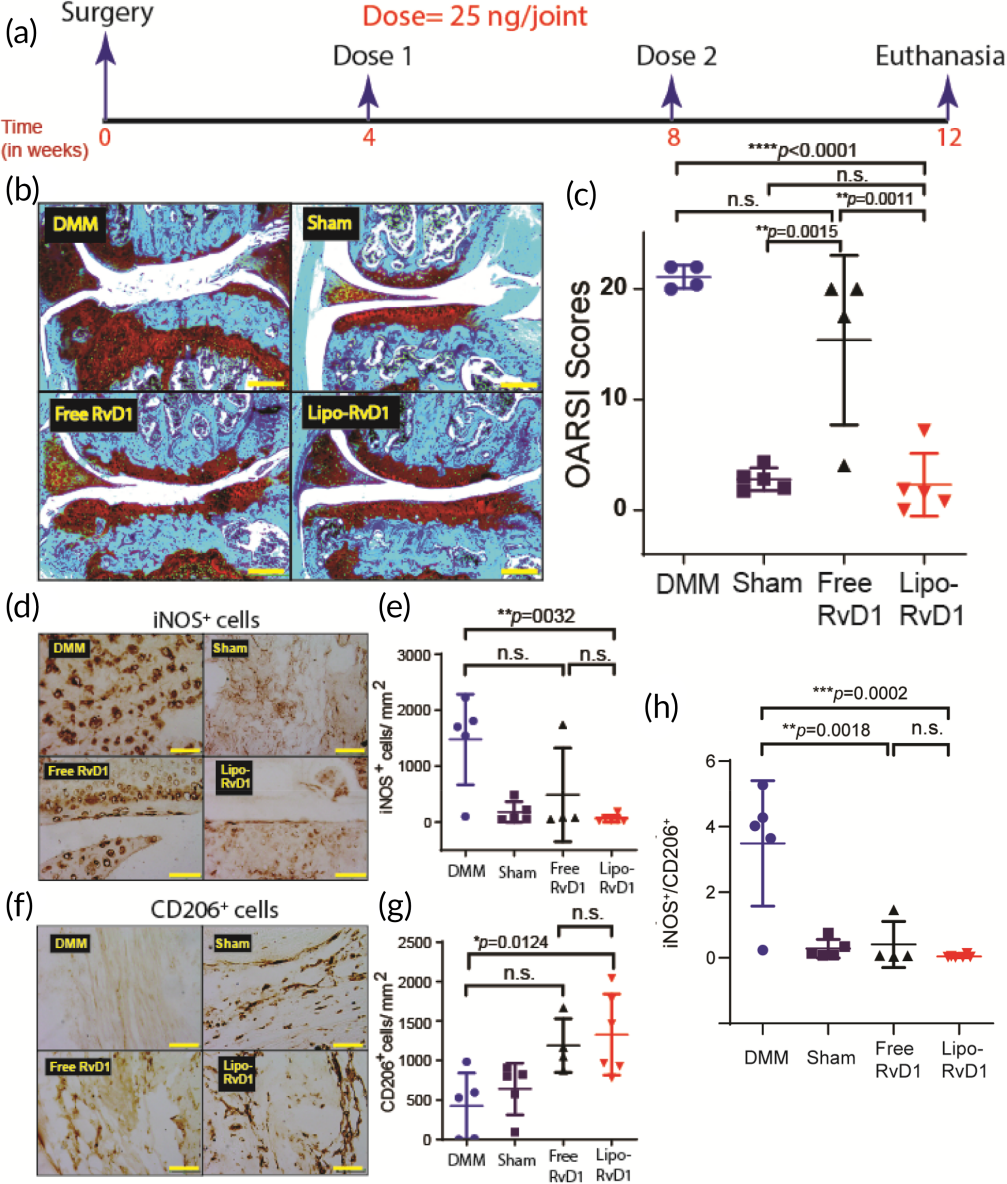
Therapeutic administration of Lipo‐RvD1 protects cartilage from progressing damage. (a) Timeline for the study. (b) Safranin O‐stained characteristic histological images of different groups of animals (scale bar = 200 μm). (c) OARSI scores of Safranin O‐stained sections of mice joints administered with respective treatment; *n* = 5 sham control joints and DMM joints, *n* = 4 joints treated with free RvD1, and *n* = 6 joints treated with Lipo‐RvD1. (d) Characteristic IHC images depicting levels of iNOS^+^ M1 macrophages in the synovial membrane of respective mice joints (scale bar = 50 μm). (e) Quantification of iNOS^+^ M1 macrophages in the synovial membrane of respective mice joints; *n* = 4 sham control joints and joints treated with free RvD1, *n* = 6 DMM joints and joints treated with Lipo‐RvD1. (f) IHC images depicting levels of CD206^+^ M2 macrophages in synovial membrane (scale bar = 50 μm). (g) Quantification of CD206^+^ M2 macrophages in the synovial membrane; *n* = 4 sham control joints and joints treated with free RvD1, *n* = 5 DMM joints, and *n* = 6 joints treated with Lipo‐RvD1. (h) The ratio of M1/M2 cells in the synovial membranes of knee joints administered with respective injections; *n* = 4–6 animals per group. For c, e, g, and h, *p < 0.05, **p < 0.01, and ****p < 0.0001, between the respective groups indicated in the figures using ANOVA followed by Tukey's posthoc test. Values are expressed as mean ± *SD*. ANOVA, analysis of variance; DMM, destabilization of the medial meniscus; IHC, immunohistochemistry

Often, the clinical symptoms of OA are seen after the disease has progressed substantially. To test Lipo‐RvD1 therapeutic efficacy, we designed a treatment regimen where our liposomal formulation was administered 4 weeks after the surgery (Figure 4a). We chose this timeline as it has been previously shown that OA‐like changes in cartilage begin to appear within 2 weeks after the DMM surgery.^45^, ^77^ We also reduced the number of IA interventions (two administrations instead of three administrations given for prophylactic treatments). In this challenging model, free‐RvD1 was ineffective as a therapeutic agent and had considerable cartilage damage (as seen from Safranin O‐stained sections and OARSI scores; Figure 4b,c). On the contrary, IA Lipo‐RvD1 administration was much more effective than free RvD1 (*p* = 0.0011) and DMM mice (*p* < 0.0001) in maintaining cartilage health and had OARSI scores similar to animals undergoing sham surgery (Figure 4b,c).

Similar to the prophylactic study, Lipo‐RvD1 treatment decreased the levels of proinflammatory M1 macrophages in the synovial membrane (*p =* 0.0032) (Figure 4d,e) while simultaneously increasing the levels of proresolution M2 macrophage (*p =* 0.0124) (Figure 4f,g) compared to DMM joints. Besides, our results also show that administration of Lipo‐RvD1 in a therapeutic regime can decrease the ratio of M1/M2 cells in the synovial membrane compared to DMM joints (*p =* 0.0002) (Figure 4h). Catabolic enzymes like MMP13 and ADAMTS5, which are known to be major drivers of cartilage damage,^78^, ^79^ were also upregulated in DMM mice cartilage, as seen in IHC images (Figure S11). We observed that the RvD1 treatment reduced the expression of these damaging enzymes and protected cartilage from degradation.

### 3.6 Lipo‐RvD1 reduces the incidence of osteophytes and OA‐associated allodynia

**FIGURE 5:**
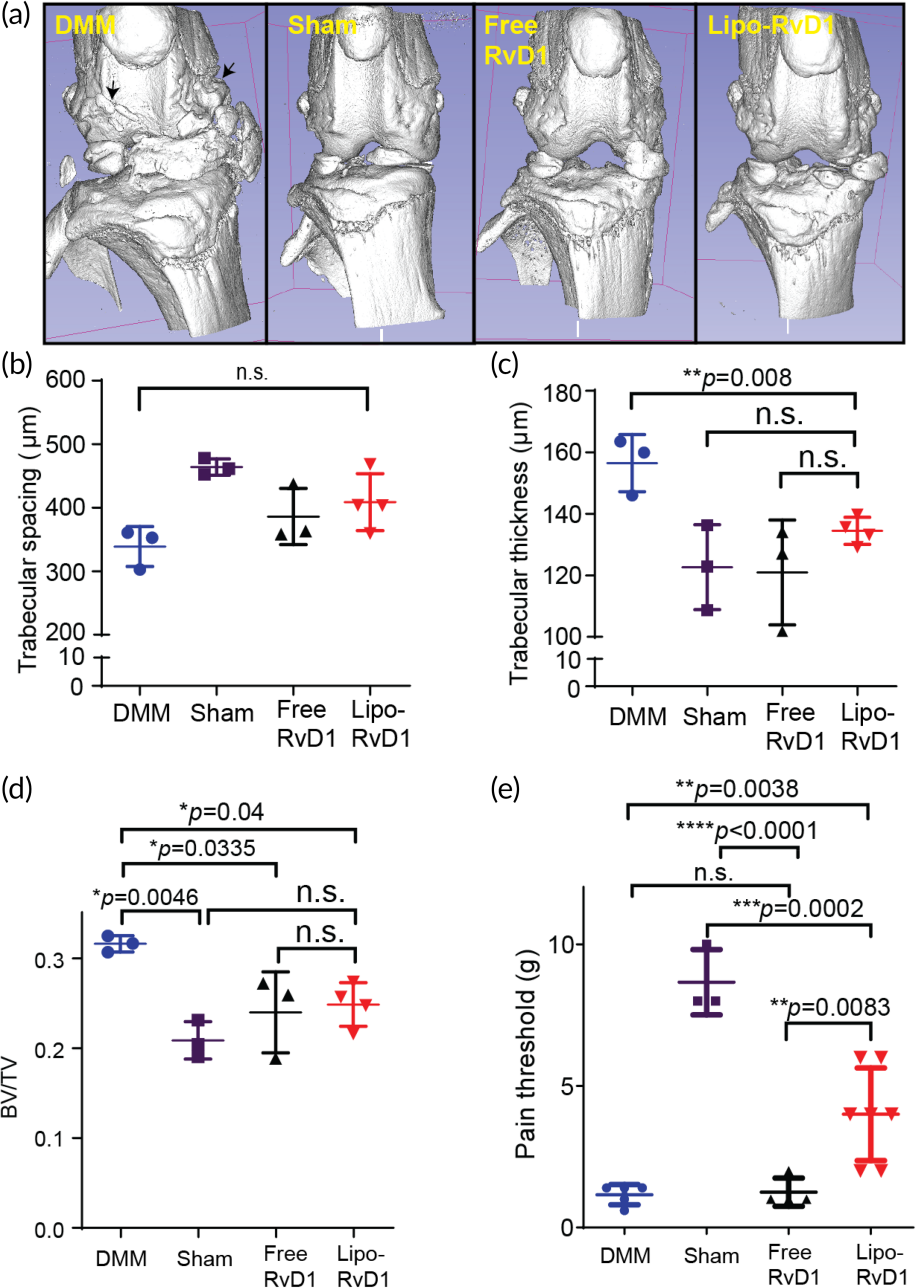
Lipo‐RvD1 reduces osteophytes and OA‐associated pain. (a) Characteristic microCT images of mice knee joints administered with respective treatments. Quantification of (b) trabecular spacing, (c) trabecular thickness, and (d) percent bone volume for different treatment groups. For b–d, *n* = 3 DMM joints, sham control joints and joints treated with free RvD1 each, and *n* = 4 joints treated with Lipo‐RvD1. (e) Paw‐withdrawal response of different treatment groups as measured by von Frey filaments. *n* = 5 for DMM joints and sham control, *n* = 7 for Lipo‐RvD1‐treated joints, and *n* = 4 for free RvD1‐treated joints. For b–e, *p < 0.05, **p < 0.01, ***p < 0.001, and ****p < 0.0001 between the respective groups indicated in the figures using ANOVA followed by Tukey's posthoc test. Values are expressed as mean ± *SD*. ANOVA, analysis of variance; DMM, destabilization of the medial meniscus; OA, osteoarthritis

Next, we analyzed the effect of Lipo‐RvD1 on two major clinical symptoms associated with OA: osteophytes and pain. Our microCT data showed that surgically induced OA increased bony growth in the joint, which was inhibited by both free and Lipo‐RvD1 (Figure 5a). Subchondral bone was analyzed for trabecular thickness, spacing, and bone volume versus total volume. Both free and Lipo‐RvD1 treatments prevented calcification of ectopic trabecular structures and showed improvement of the parameters BV/TV (*p* = 0.04) and trabecular thickness (*p =* 0.008) when compared against DMM joint (Figure 5b–d). This shows the potential of RvD1 in the treatment of OA.

Pathological pain (allodynia) is one of the main clinical symptoms of OA.^80^ To test if resolvin formulations decreased pain, we tested the pain threshold of mice using von Frey filaments. We observed that administration of lipo‐RvD1 was effective in alleviating the allodynia than DMM mice (*p* = 0.0038) and free RvD1 injected mice (*p* = 0.0083) (Figure 5e). IA injection of free RvD1 did not generate sufficient analgesia, and the pain threshold of these mice was similar to that in DMM‐operated mice (Figure 5e). While DMM‐operated and free drug administered group had a low pain threshold (<2 g), Lipo‐RvD1 injected animals showed close to 4 g, which was closer to sham controls. This analgesic effect of Lipo‐RvD1 was not present by Day 8 postinjection. The exact source of OA‐related allodynia is not known, but the transient receptor potential (TRP) family of mediators is known to play a critical role in response to mechanical stimuli, including those inducing pain.^81^ Members of this family, especially TRPV1 and TRPV4, are associated with the severity of pain in OA.^82^, ^83^, ^84^ RvD1 has been shown to have an antinociceptive effect by targeting members of this family, especially TRPV3, TRPV4, and TRPA1.^85^, ^86^ The role of RvD1 receptors in analgesia is demonstrated earlier.^87^ Our study shows for the first time that the sustained presence of RvD1 in the affected knee joint can help alleviate OA‐associated allodynia after injection but fails to maintain this effect in the long term (>7 days). This could be possibly due to reduced joint concentrations of RvD1 after a few days of injection. The pain relief could be important translationally as not only would it provide immediate benefit, but would also ensure patient compliance.

While our Lipo‐RvD1 has shown promising results, mice knee joints are small and can tolerate IA injections of only 2–10 μl.^88^ The mice cartilage is 70 times thinner, and the load is also substantially lower than biped joints such as humans.^89^ Hence, for future translation, further testing is required in larger animals.

## 4 DISCUSSION

OA is the most common joint disease and is associated with chronic low‐grade inflammation. The administration of ω‐3 fatty acids are long known to have an anti‐inflammatory effect.^90^ These molecules are often pleiotropic and target multiple pathways. Studies have shown that molecules such as docosahexaenoic acid and eicosapentaenoic acid are non toxic and have shown promise in several chronic inflammatory diseases, including OA.^91^, ^92^, ^93^ RvD1 is one such species of ω‐3 fatty acids that have shown a remarkable potential to reduce inflammation in many chronic inflammatory diseases,^94^, ^95^ including OA.^29^, ^87^ However, due to low molecular weight, these drugs are rapidly cleared and limits the clinical translation.

Biomaterials loaded with the active molecules act as a depot that releases the therapeutic concentrations of cargo in a controlled manner.^15^, ^36^, ^50^ Resolvin E1 delivery via polymeric nanoparticles was successfully used to treat wounds in a mice model of intestinal injury.^53^ RvD1 successfully reduced the influx of neutrophils after delivery via poly‐l‐(lactic‐co‐glycolic acid) scaffolds.^52^ While successful, these strategies are not amenable for IA delivery due to low encapsulation efficiencies or large size.

Liposomes are made of inert, biocompatible lipids with tunable drug release properties and were the first nanocarriers to be approved for use in humans.^96^ In this study, we developed an RvD1 encapsulating liposomal formulation that shows tunable and high loading and releases RvD1 over ~11 days. This is the first study that demonstrates a tunable and controlled release of an SPM over a prolonged duration using injectable nanocarriers. The rationale for designing such a carrier was to stabilize the drug in vivo and increase the availability of the drug for longer durations.

One of the hallmarks of OA is heightened sensitivity to pain.^97^, ^98^ The TRP family of mediators is known to play a critical role in response to mechanical stimuli, including those inducing pain.^81^ Members of this family, especially TRPV1 and TRPV4, are associated with the severity of pain in OA.^82^, ^83^, ^84^ RvD1 has been shown to have an antinociceptive effect by targeting members of this family, especially TRPV3, TRPV4, and TRPA1.^85^, ^86^ The superiority of RvD1 as an analgesic can be emphasized by its ability to target central sensitization.^99^ The role of RvD1 receptors in analgesia is demonstrated earlier.^87^ Our study shows for the first time that the presence of RvD1 in the affected knee joint can help alleviate OA‐associated allodynia after injection but fails to sustain this effect in the long term (>7 days). This could be possibly due to reduced joint concentrations of RvD1 after a few days of injection. The pain relief could be important translationally as not only it would provide immediate benefit, it would also ensure patient compliance.

Macrophages are one of the major players of OA and can be divided into two phenotypes—M1 (proinflammatory) and M2 (anti‐inflammatory). It has been shown that the balance between these two populations is critical for homeostasis, and an imbalance is often seen in inflammatory diseases.^73^, ^74^ The ratio of M1/M2 cells is skewed in OA, and proinflammatory cytokines from M1 drive cartilage damage.^75^ Previously, it was shown that RvD1, via its action on ALX/FPR2 receptor, promotes the polarization of macrophages toward the M2 phenotype. However, the treatment regimen included administration of drug before surgery and any symptoms.^29^ RvD1‐liposomes provides a translatable regimen by administering the formulation after induction of trauma (prophylactic dosing) or when significant damage is incurred to the joint (therapeutic dosing).

While our Lipo‐RvD1 has shown promising results, mice knee joints are small and can tolerate IA injections of only 2–10 μl.^88^ The mice cartilage is 70 times thinner, and the load is also substantially lower than biped joints such as humans.^89^ Hence, for future translation, further testing is required in larger animals.

## 5 CONCLUSION

In this study, we formulated nanoliposomes that can arrest the deterioration of the cartilage in mice models of OA at a much lower dose than reported earlier. Controlled release of RvD1 was achieved for more than 11 days. Lipo‐RvD1 increased the proportion of M2 cells in the synovium and promoted the resolution of inflammation resulting in substantial lower damage to cartilage. This formulation also reduced OA symptoms, including ectopic bone formation, cartilage degradation, and OA‐associated pain. Lipo‐RvD1 can be administered both as a prophylactic and therapeutic treatment and can be a promising strategy to treat OA.

## AUTHOR CONTRIBUTIONS

**Ameya Atul Dravid:** Investigation (lead); methodology (lead); writing – original draft (lead); writing – review and editing (equal). **Kaamini Dhanabalan:** Methodology (supporting); writing – original draft (supporting); writing – review and editing (supporting). **Smriti Agarwal:** Methodology (supporting); writing – original draft (supporting); writing – review and editing (supporting). **Rachit Agarwal:** Conceptualization (lead); funding acquisition (lead); investigation (supporting); methodology (supporting); supervision (equal); writing – original draft (supporting); writing – review and editing (equal).

## CONFLICT OF INTERESTS

The authors do not declare any conflicting interests.

### PEER REVIEW

The peer review history for this article is available at https://publons.com/publon/10.1002/btm2.10281.

## Supporting information


**Appendix** **S1:** Supporting Information

## Data Availability

The data that support the findings of this study are available from the corresponding author upon reasonable request.
