# Supplementary material for: Resolvin D1‐loaded nanoliposomes promote M2 macrophage polarization and are effective in the treatment of osteoarthritis
Source: Bioeng Transl Med. 2022 Mar 7;7(2):e10281. doi: 10.1002/btm2.10281 (PMC9115708; doi:10.1002/btm2.10281)
Supplement: Supplementary file 1 — Appendix S1: Supporting Information [file BTM2-7-e10281-s001.docx]

Supporting Information

**Resolvin D1-loaded nanoliposomes promote M2 macrophage polarization and are effective in** **treatment of Osteoarthritis**

Author(s), and Corresponding Author(s)*

*Ameya A. Dravid^a^, Kaamini M. Dhanabalan^a^, Smriti Agarwal^a^, Rachit Agarwal^a,^**


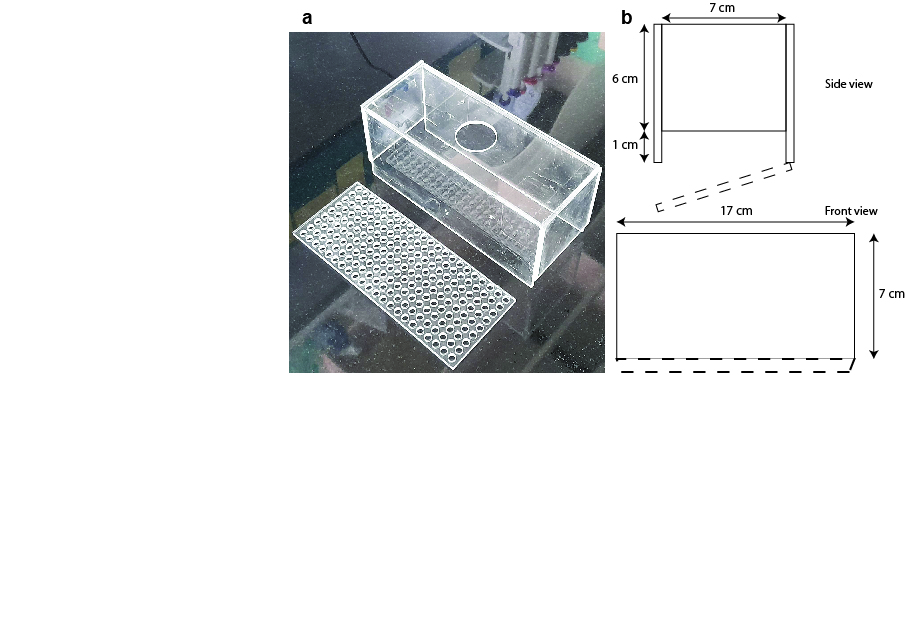


**Figure S1. Design of enclosure for von Frey testing*.*** (**a**) Representative photo of the enclosure that allows free movement of mice and convenient access to plantar regions of the limbs from the bottom. (**b**) Front and side view of the enclosure depicting the dimensions of the enclosure.





**Figure S2. Sizes of liposomes were maintained in PBS*.*** Size stability of liposomes in PBS as measured by DLS; n=3. Data were represented as mean ± SD.


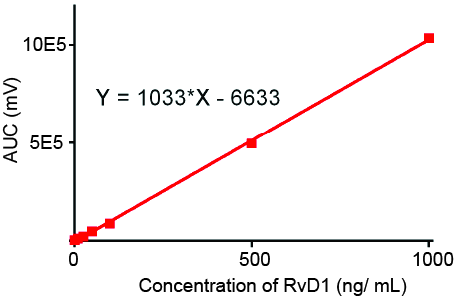


**Figure S3. The standard curve for quantification of RvD1 was generated using HPLC.** Plot of the area under the corresponding peak on the chromatogram (area under the curve; AUC) against concentration of RvD1 injected. n=2 replicates for each concentration.


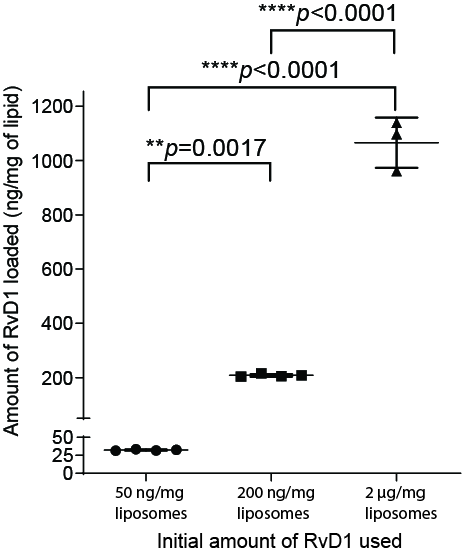


**Figure S4. Loading achieved for lipo-RvD1 was tunable*.*** Plot depicting RvD1 loaded as a function of initial gradient provided across the lipid bilayer of the liposomes; n=4 replicates for liposomes loaded with 50 ng/mg and 200 ng/mg of lipids and n=3 replicates for liposomes loaded with 2000 ng/mg lipid. *****p* <0.0001 between all groups determined using ANOVA followed by Tukey’s posthoc test. Data were represented as mean ±SD.


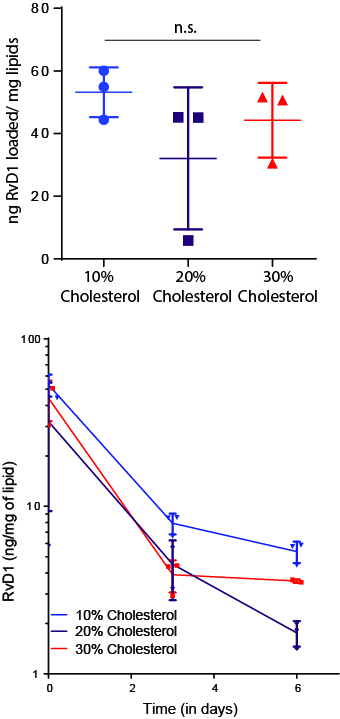


**Figure S5. Liposomal cholesterol does not affect RvD1 loading.** Loading of RvD1 into liposomes containing different cholesterol concentrations; n=3 for each liposome test group. Groups were tested for statistical significance using ANOVA. Data were represented as mean ± SD.


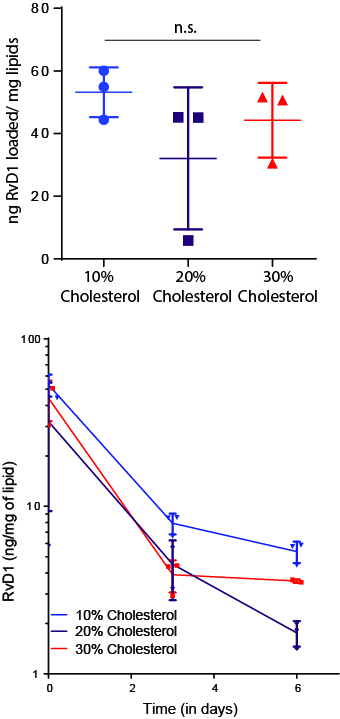


**Figure S6. Low cholesterol-containing liposomes retain RvD1 longer*.*** Retention profile of RvD1 in liposomes containing different cholesterol concentrations; n=2 replicates for day 3 of 20% cholesterol and day 6 of 30% cholesterol, and-n=3 replicates for every other time point in every group*.* Data were represented as mean ±SD.


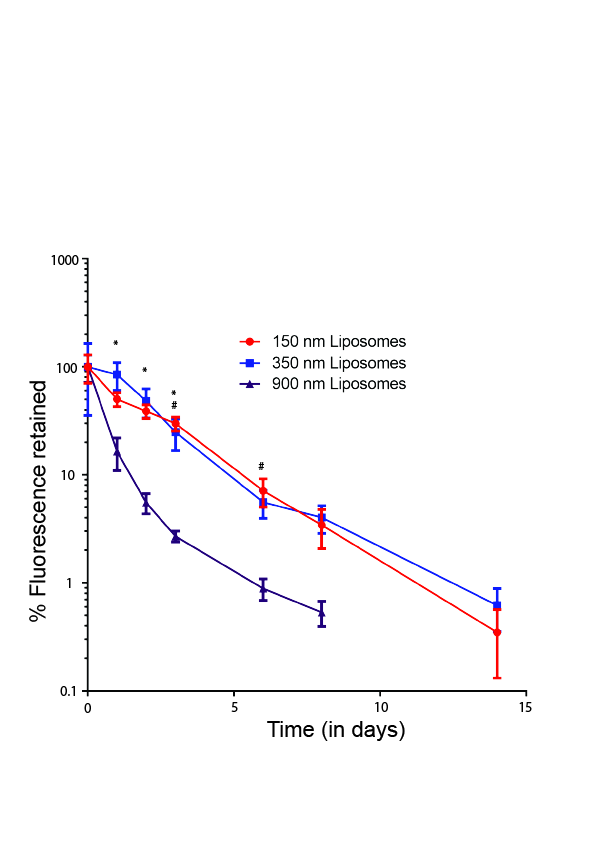


**Figure S7. Liposomes show size-dependent IA retention.** Quantification of IA clearance of different sizes of liposomes as measured by Bruker Xtreme II; n=4 injected knee joints per group for every time point. **p* <0.05 between respective data from 350 and 900 nm-liposomes using ANOVA followed by Tukey’s test. ^#^*p* <0.05 between respective data from 150 and 900 nm-liposomes using ANOVA followed by Tukey’s test. Data were represented as mean ±SEM.


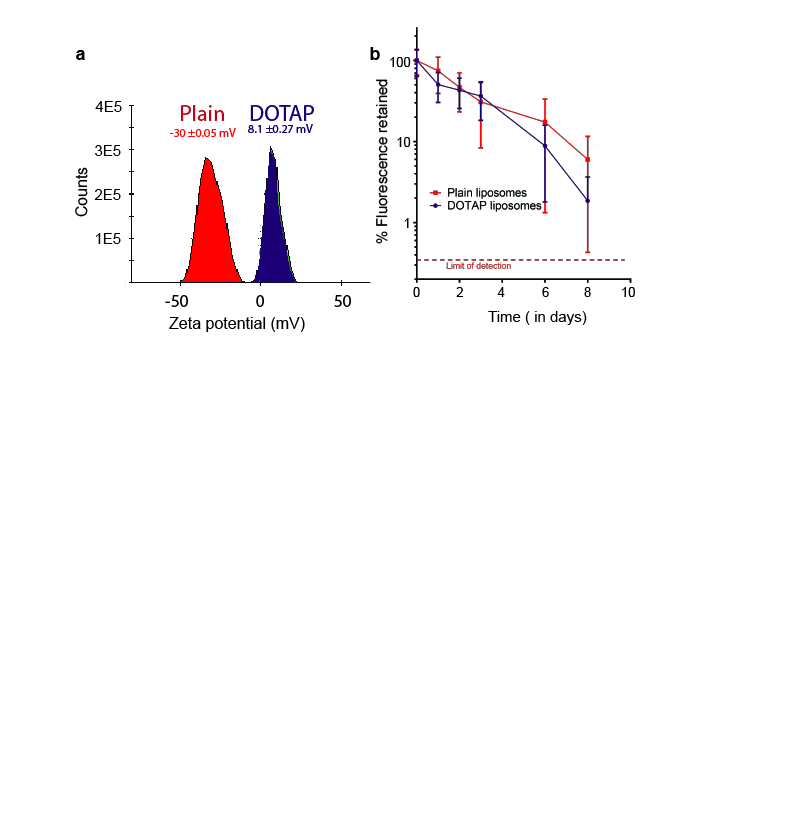


**Figure S8. Cationic liposomes do not retain longer than unmodified liposomes IA*.*** (**a**) Zeta potential of cationic and plain liposomes. (**b**) Comparison between IA temporal retention of plain and cationic liposomes in mice knee joint after respective intraarticular injection; n=6 injected knee joints per group for every time point. Data were represented as mean ± SEM.


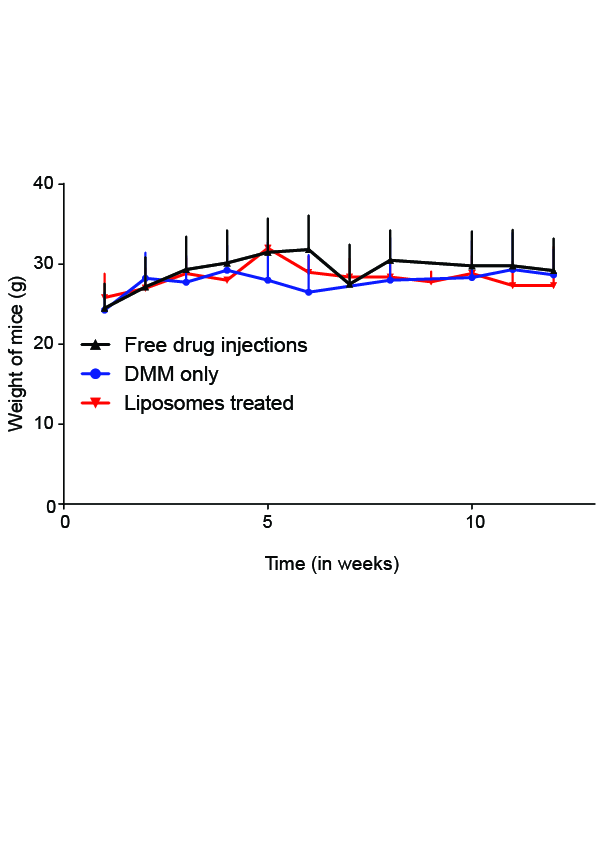


**Figure S9. IA injections of liposomes do not affect the general conditioning of mice.** Plot of weights of animals with respect to time; n=4 DMM-only mice and n=6 mice for other groups. Data were represented as mean ±SD.


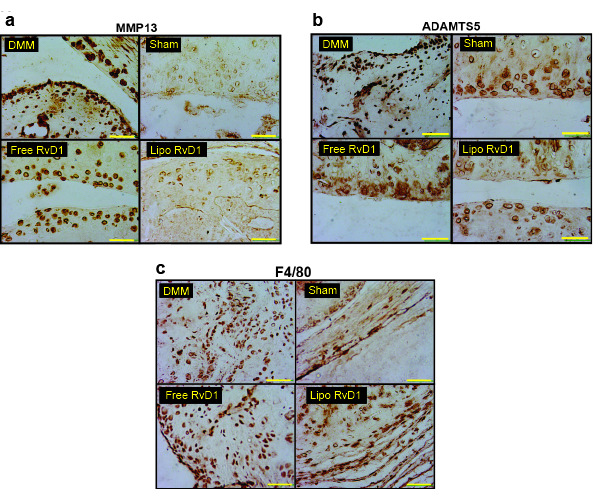


**Figure S10.** **Prophylactic administration of lipo-RvD1 inhibits the activity of catabolic mediators.** Representative IHC images of synovial membrane stained for (**a**) matrix metalloproteinase 13 (MMP13) and (**b**) A disintegrin and metalloproteinase with thrombospondin motifs-5 (ADAMTS5). Scale bar 50 µm.


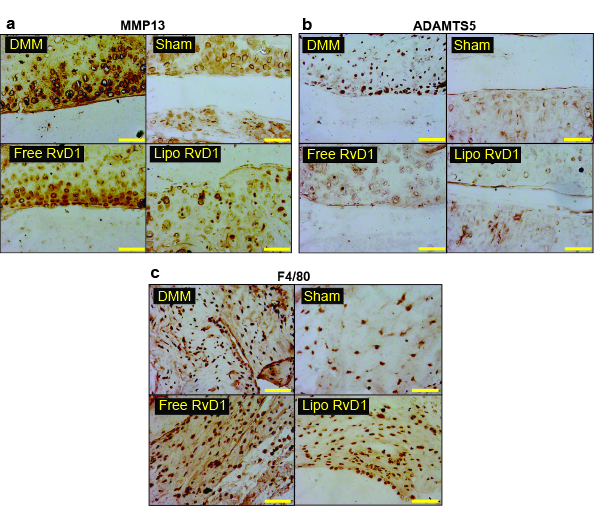


**Figure S11.** **Therapeutic administration of lipo-RvD1 inhibits the activity of catabolic mediators in OA joints.** Representative IHC images of synovial membrane stained for (**a**) matrix metalloproteinase 13 (MMP13) and (**b**) A disintegrin and metalloproteinase with thrombospondin motifs-5 (ADAMTS5). Scale bar 50 µm.
